# Checklist of known moth flies and sand flies (Diptera, Psychodidae) from Armenia and Azerbaijan

**DOI:** 10.3897/zookeys.798.26543

**Published:** 2018-11-21

**Authors:** Jan Ježek, Peter Manko, Jozef Oboňa

**Affiliations:** 1 Department of Entomology, Cirkusová 1740, CZ – 193 00 Praha 9 – Horní Počernice, Czech Republic Department of Entomology, National Museum Prague Czech Republic; 2 Department of Ecology, Faculty of Humanities and Natural Sciences, University of Prešov, 17. novembra 1, SK – 081 16 Prešov, Slovakia University of Prešov Prešov Slovakia

**Keywords:** Caucasus, checklist, Diptera, faunistics, first records, Phlebotominae, Psychodidae, Psychodinae

## Abstract

All credible and available published records for 17 species of moth flies known so far from Armenia (Phlebotominae 11 species, Psychodinae 6 species) and Azerbaijan (Phlebotominae 18 species) are summarized. The first records of 18 species of Psychodinae (tribes Mormiini, Paramormiini, Psychodini, Pericomaini) from Armenia and 6 new faunistic records (tribes Psychodini, Pericomaini) for the fauna of Azerbaijan are listed. The checklist of recent moth flies from Armenia includes now 35 species, and from Azerbaijan, 24 species.

## Introduction

As mentioned by Oboňa et al. (2017b), the Caucasus is among the top 25 biologically richest and most endangered hotspots in the world, with an exceptional concentration of endemic species and species at risk. It is also one of the ecoregions of WWF’s Global 200 project, which were identified as having outstanding terrestrial, freshwater, and marine habitats (Myers et al. 2000; Krever et al. 2001). The remarkable richness of the flora and fauna is determined by complex orography, geology, and climate and results in a variety of habitats, landscapes, and microclimates in this mountain range at the border of Europe and Asia and at the junction of temperate and subtropical zones. This area is affected by both Atlantic air masses and the dry continental climate of continental Eurasia (Price 2000).

The location of this area creates favourable conditions for entomological research. However, several families of flies have not been well studied in the Caucasus, and in particular, in Armenia and Azerbaijan (Oboňa et al. 2016b, 2017b).

Moth flies (Psychodidae) are represented only by 17 species previously recorded in Armenia (mainly Perfil’ev 1966; Lewis 1982; Artemiev and Neronov 1984; Vaillant 1972; Wagner 1981, 1990, 2013; Wagner and Joost 1986; Wagner et al. 2002; Melaun et al. 2014; Oboňa et al. 2017a). These species belong to subfamilies Phlebotominae [11 species: Phlebotomus (P.) papatasi (Scopoli, 1786); P. (Paraphlebotomus) alexandri Sinton, 1928; P. (P.) cucasicus Marzinovsky, 1917; P. (P.) sergenti Parrot, 1917; P. (P.) similis Perfil’ev, 1963; P. (Larroussius) kandelakii Shurenkova, 1929; P. (L.) tobbi Adler & Theodor in Adler, Theodor & Lourie, 1930; P. (Adlerius) halepensis Theodor, 1958; P. (A.) simici Nitzulescu, 1931; Sergentomyia (Neophlebotomus) pawlowskyi (Perfiliev, 1933); and S. (Parrotomyia) palestinensis (Adler & Theodor, 1927)] and Psychodinae [6 species: *Parajungiellamonikae* (Wagner & Joost, 1986); Paramormia (P.) fratercula (Eaton, 1893); *Joostiellacaucasica* Vaillant, 1983; Pericoma (P.) exquisita Eaton, 1893; *Pneumiajoosti* (Wagner, 1981); and *Thornburghiellaveve* Oboňa & Ježek, 2017].

From Azerbaijan, 18 species of Psychodidae have been recorded, all belonging to subfamily Phlebotominae [Phlebotomus (P.) papatasi (Scopoli, 1786); P. (Paraphlebotomus) alexandri Sinton, 1928; P. (P.) cucasicus Marzinovsky, 1917; P. (P.) jacusieli Theodor, 1947; P. (P.) mongolensis Sinton, 1928; P. (P.) sergenti Parrot, 1917; P. (P.) similis Perfil’ev, 1963; P. (Larroussius) kandelakii Shurenkova, 1929; P. (L.) perfiliewitranscaucasicus Perfiliew, 1937; P. (L.) perniciosus Newstead, 1911; P. (L.) tobbi Adler & Theodor in Adler, Theodor & Lourie, 1930; P. (Adlerius) balcanicus Theodor, 1958; P. (A.) brevis Theodor & Mesghali, 1964; P. (A.) halepensis Theodor, 1958; P. (A.) simici Nitzulescu, 1931, Sergentomyia (S.) dentata Sinton, 1933; S. (Neophlebotomus) pawlowskyi (Perfiliev, 1933); and S. (Parrotomyia) palestinensis (Adler & Theodor, 1927)].

## Material and methods

The material presented here comes from two different sampling campaigns. The first campaign collected material by sweep-netting vegetation along streams and lakes in Armenia from August 26 to September 4, 2015 by J. Oboňa, P. Manko and Ľ. Hrivniak; it is preserved in 75% ethanol. A list of 28 sampling sites, with coordinates and altitudes, is given in Table 1. Captured moth flies were mounted in Canada balsam on 85 slides in the laboratory. The second collection campaign by Ľ. Hrivniak in Azerbaijan from May 26 to June 4, 2017 used the same collecting methods. Samples were collected at various sites by sweep-netting from vegetation along streams and lakes and light trapping by H. A list of 4 sampling sites, with coordinates and altitudes, is given in Table 2. The collected material was preserved in 96% ethanol in the field. Captured moth flies were mounted in Canada balsam on 7 slides in laboratory.

All material, determined by the first author, is deposited in the National Museum, Natural History Museum, Department of Entomology, Prague, Czech Republic. Slides are numbered by inventory slide number of the family Psychodidae, and catalogue number (cat. no.) of the slide to be included in the NMPCDiptera collection (Tkoč et al. 2014). Nomenclature is according to Perfil’ev (1966), Lewis (1982), and Artemiev and Neronov (1984) for Phlebotominae; for Psychodinae the nomenclature is modified from Vaillant (1972) and Wagner (1990, 2013) using the classifications of e.g. Ježek and van Harten 2005, Oboňa and Ježek (2014), and Kroča and Ježek (2015). The following abbreviations are used in the paper: H = Ľ. Hrivniak leg., J = J. Ježek det., Ma = P. Manko leg., O = J. Oboňa leg., NMPC = collections of the National Museum Prague, HC = hand collecting, SW = sweep netting, LT = light traps.

**Table 1.** List of sampling sites in Armenia.

Site no.	site name (province, short description of locality)	Latitude (N)/ Longitude (E)	Altitude (m) a.s.l.
Arm 1	Yerevan, Tigran Mets Avenue	40°10'19.6"N, 44°30'49.1"E	976
Arm 2	Kotayk Province, Marmarik district, Marmarik Secondary School	40°35'04.0"N, 44°40'10.1"E	1750
Arm 3	Kotayk Province, Marmarik distr., Marmarik near roud H28	40°35'04.0"N, 44°40'10.1"E	1750
Arm 4	Kotayk Province, Marmarik River, below Hankavan	40°38'04.7"N, 44°29'19.4"E	1974
Arm 5	Kotayk Province, Hrazdan River, above Solak town	40°28'19.7"N, 44°42'42.2"E	1567
Arm 6	Kotayk Province, Hrazdan River, below Hrazdan Reservoir	40°29'12.8"N, 44°43'55.9"E	1705
Arm 7	Kotayk Province, tributary of Marmarik River, above Meghradzor, behind railway	40°37'12.7"N, 44°40'18.3"E	1825
Arm 8	Kotayk Province, tributary of the Marmarik River, above recreation center	40°33'52.0"N, 44°40'09.1"E	1872
Arm 9	Tavush Province, Lake Parz Lich and its tributary, lake Parz Lich	40°44'57.7"N, 44°57'33.3"E	1376
Arm 10	Tavush Province, Bldan River, above the Dilijan City	40°44'49.1"N, 44°49'03.5"E	1354
Arm 11	Tavush Province, Bldan River, below Jukhtakvank monastery and the mineral water factory/plant	40°45'11.8"N, 44°48'25.7"E	1411
Arm 12	Tavush Province, tributary of Aghstev River, above Teghut town	40°48'09.3"N, 44°53'43.7"E	1382
Arm 13	Gegharkunik Province, Dzknaget River, at Sevan Lake and road M14	40°37'01.8"N, 44°57'44.2"E	1930
Arm 14	Ararat Province, Gekhard River, at Gerghard monastery (parking place)	40°11'03.2"N, 44°31'18.6"E	1770
Arm 15	Ararat Province, Gekhard River, below Garni Temple	40°08'24.7"N, 44°49'04.2"E	1240
Arm 16	Ararat Province, small tributary of Azat River, waterfall at road	40°06'33.2"N, 44°43'49.3"E	1249
Arm 17	Ararat Province, above the confluence of Azat and Gekhard rivers	40°06'39.4"N, 44°43'45.3"E	1273
Arm 18	Ararat Province, small tributary of Gekhard River, crossroad at the factory	40°07'00.4"N, 44°44'35.7"E	1340
Arm 19	Ararat Province, Azat River, at Lanjazat village	40°03'27.0"N, 44°34'38.3"E	976
Arm 20	Lori Province, tributary of the Aghstev River, above M8 road at Lermontov village	40°45'24.6"N, 44°38'42.0"E	1853
Arm 21	Lori Province, Zamanlu River, a tributary of Pambak River, at Vahagnadzor town	40°53'07.0"N, 44°34'39.0"E	1092
Arm 22	Lori Province, tributary of Pambak River, at the H24 road switch-backs	40°56'52.7"N, 44°37'37.2"E	1030
Arm 23	Lori Province, small steppe brook, tributary of Dzoraget River	41°03'59.9"N, 44°05'44.2"E	1949
Arm 24	Shirak Province, tributary of Akhurian River, in valley below road from above Amasia town	40°58'20.5"N, 43°46'06.9"E	1987
Arm 25	Shirak Province, tributary of Akhurian River, at Torosgyugh village	40°55'55.0"N, 43°52'45.3"E	1885
Arm 26	Lori Province, small brook, in valley at the road H23 to Pushkin Pass	40°54'22.9"N, 44°25'33.3"E	1839
Arm 27	Lori Province, tributary of the Dzoraget River, above Pushkin village	40°58'04.8"N, 44°24'49.7"E	1485
Arm 28	Tavush Province, tributary of the Gosh River, spring area at parking place and cafeteria	40°44'15.9"N, 45°01'01.2"E	1039

**Table 2.** List of sampling sites in Azerbaijan.

Site no.	Site name (province, short description of locality)	Latitude (N)/ Longitude (E)	Altitude (m) a.s.l.
Aze 1	Khizi district, S of Sitalçay, wetland/pasture near Sumgayit bypass highway	40°40'38.4"N, 49°29'08.9"E	169
Aze 2	Quba district, Xinaliq village, mountain stream	41°11'00.3"N, 48°07'42.9"E	2170
Aze 3	Lankaran district, SW of Lankaran, stream with woody vegetation, tributary of Lankaran River	38°42'59.2"N, 48°44'17.8"E	75
Aze 4	Qabala district, S of Durca, light trap near stream, tributary of Qaraschay River	41°02'11.2"N, 47°53'13.6"E	1236

## Results

### Psychodidae

#### Phlebotominae

##### *Phlebotomus* Rondani, 1840 (sensu Sabrosky, 1999)

###### Subgenus Phlebotomus s. str.

**1. Phlebotomus (Phlebotomus) papatasi (Scopoli, 1786)**

**Selected published records.**Perfil’ev (1966: 252–254); Lewis (1982: 138–140); Artemiev and Neronov (1984: 39, 40); Wagner (1990: 13); Wagner et al. (2002: 323); Melaun et al. (2014: 2299).

**Distribution.** Afghanistan, Albania, Algeria, Armenia, Azerbaijan, Baleares, Bosnia and Herzegovina, Bulgaria, Crete, Crimea, Croatia, Cyprus, Egypt, Ethiopia, France, Georgia, Greece, Hungary, India, Iran, Iraq, Israel, Italy, Jordan, Kazakhstan (southern), Kuwait, Libya, Macedonia, Malta, Moldova, Montenegro, Morocco, Oman, Pakistan, Portugal, Romania, Sardinia, Saudi Arabia, Serbia, Slovenia, Spain, Sudan, Syria, Tunisia, Turkey, Ukraine (southern), Yemen.

###### Subgenus Paraphlebotomus Theodor, 1948

**2. Phlebotomus (Paraphlebotomus) alexandri Sinton, 1928**

**Selected published records.**Perfil’ev (1966: 264–267); Lewis (1982: 143); Artemiev and Neronov (1984: 45); Wagner (1990: 14); Melaun et al. (2014: 2298).

**Distribution.** Afghanistan, Albania, Algeria, Armenia, Azerbaijan, Bulgaria, China (western), Crimea, Cyprus, Djibouti, Ethiopia, Georgia, Greece, India, Iran, Iraq, Israel, Kazakhstan (southern), Moldova, Mongolia, Morocco, Pakistan, Romania, Saudi Arabia, Spain, Sudan, Tunisia, Turkey, Ukraine, United Arab Emirates, Yemen; northern Sahara, Caucasus (southern), Near and Middle East, Central and Eastern Asia; Afrotropical and Oriental regions.

**3. Phlebotomus (Paraphlebotomus) cucasicus Marzinovsky, 1917**

**Selected published records.**Perfil’ev (1966: 257–261, as *grimmi* Porchinskyi, 1876); Lewis (1982: 144–145, as *caucasicus*); Artemiev and Neronov (1984: 49, as *caucasicus*); Wagner (1990: 14, as *caucasicus*); Melaun et al. (2014: 2298, as *caucasicus*).

**Distribution.** Afghanistan, Armenia, Azerbaijan, Bulgaria, China (not verified), Georgia, Greece, Iran, Kazakhstan, Macedonia, Turkmenistan, Uzbekistan.

**4. Phlebotomus (Paraphlebotomus) jacusieli Theodor, 1947**

**Selected published records.**Perfil’ev (1966: 110, distribution only); Lewis (1982: 145); Artemiev and Neronov (1984: 50); Wagner (1990: 14); Melaun et al. (2014: 2298).

**Distribution.** Albania, Azerbaijan, Cyprus, Greece, Israel, Jordan, Iran, northern Palestine, Turkey.

**5. Phlebotomus (Paraphlebotomus) mongolensis Sinton, 1928**

Syn. *Phlebotomusimitabilis* Artemiev, 1974 (see Artemiev 1978: 18).

**Selected published records.**Perfil’ev (1966: 267–271); Lewis (1982: 146); Artemiev and Neronov (1984: 55–56); Wagner (1990: 14); Melaun et al. (2014: 2299).

**Distribution.** Afghanistan, Azerbaijan, China, Iran, Kazakhstan, Mongolia.

**6. Phlebotomus (Paraphlebotomus) sergenti Parrot, 1917**

**Selected published records.**Perfil’ev (1966: 274–278, only important diagnostic characters of subsp. sergenti in comparison with subsp. similis Perfil’ev, 1963); Lewis (1982: 147, recognized two mentioned subspecies); Artemiev and Neronov (1984: 57, recognized *sergenti* and *similis* as two bona species); Wagner (1990: 14, as species); Wagner et al. (2002: 323); Melaun et al. (2014: 2299, as species).

**Distribution.** Afghanistan, Albania, Algeria, Armenia, Azerbaijan, Baleares, Bosnia and Herzegovina, Bulgaria, Canary I., China, Croatia, Cyprus, Egypt, France (Corsica), Georgia, Greece, India, Iran, Iraq, Israel, Italy, Jordan, Kazakhstan, Lebanon, Libya, Madeira, Macedonia, Mali, Malta, Morocco, Portugal, Romania, Saudi Arabia, Serbia, Slovenia, Somali Republic, Spain, Syria, Tunisia, Turkey, Ukraine, Yemen. Afrotropical and Oriental regions.

**7. Phlebotomus (Paraphlebotomus) similis Perfil’ev, 1963 (sensu Artemiev & Neronov, 1984)**

Phlebotomus (Paraphlebotomus) sergentisimilis Perfil’ev, 1963

**Selected published records.**Perfil’ev (1966: 276); Lewis (1982: 148, as *sergentisimilis*); Artemiev and Neronov (1984: 58–59, as a species).

**Distribution.**Perfil’ev (1966): southern coast of Crimea and southern Ukraine, Russia, northern Caucasus. Lewis (1982): same, Azerbaijan, Uzbekistan. Artemiev and Neronov (1984): Crimea, southern Ukraine, ?northern Caucasus, Albania, Romania, and former Yugoslavia. Melaun et al. (2014: 2299): Albania, Armenia, Azerbaijan, Bosnia and Herzegovina, Bulgaria, Croatia, Georgia, Greece, Macedonia, Romania, Serbia, Slovenia, Ukraine.

###### Subgenus Larroussius Nitzulescu, 1931

**8. Phlebotomus (Larroussius) kandelakii Shurenkova, 1929**

**Selected published records.**Perfil’ev (1966: 286, as *kandelakii*); Lewis (1982: 154, ssp. *kandelakii* and *burneyi* Lewis, 1967 recognized); Artemiev and Neronov (1984: 87, *burneyi* as a species); Wagner (1990: 15) ; Melaun et al. (2014: 2298).

**Distribution.** Afghanistan, Armenia, Azerbaijan, Dagestan, Georgia, Iran, Iraq, Lebanon, Turkey, Turkmenistan, Uzbekistan.

**9. Phlebotomus (Larroussius) majorsyriacus Adler & Theodor, 1931**

**Selected published records.**Perfil’ev (1966: 278–286, four subspecies recognized: *major*, *neglectus* Tonnoir, 1921, *syriacus* Perfil’ev, 1966 and *krimensis* Perfil’ev, 1966); Lewis (1982: 156–157, added *wui* Yang & Xiong, 1965); Artemiev and Neronov (1984: 92–95, considered *neglectus* as a separate valid species, but taxonomic position of *syriacus* is unclear due to the missing differential diagnosis in the original description); Wagner (1990: 16, recognized all four subspecies); Melaun et al. (2014: 2298, recognized *syriacus* as a species).

**Distribution.** Armenia, Azerbaijan, Crimea, Georgia, Greece (Crete), Israel, Italy (Sicily), Jordan, Lebanon, Palestine, Serbia, Syria, Turkey, Ukraine.

**10. Phlebotomus (Larroussius) perfiliewitranscaucasicus Perfiliew, 1937**

**Selected published records.**Perfil’ev (1966: 289–293, recognized three subspecies: *perfiliewi*, *transcucasicus*, and *galilaeus* Theodor, 1958); Lewis (1982: 160–161, 3 subspecies); Artemiev and Neronov (1984: 88, map 30, three subspecies); Wagner (1990: 16–17, three subspecies).

**Distribution.** Azerbaijan, Iran, Iraq, Russia, Uzbekistan.

**11. Phlebotomus (Larroussius) perniciosus Newstead, 1911**

**Selected published records.**Perfil’ev (1966: 299–300, only comments on systematic position); Lewis (1982: 161–162); Artemiev and Neronov (1984: 99–100); Wagner (1990: 17); Wagner et al. (2002: 323); Melaun et al. (2014: 2298).

**Distribution.** Albania, Algeria, Andorra, Azerbaijan, Baleares, Bosnia and Herzegovina, Bulgaria, Canary I., Corsica, Croatia, Cyprus, France, Germany, Greece, Italy, Libya, Macedonia, Malta, Morocco, Portugal, Romania, Sardinia, Serbia, Sicily, Slovenia, Spain, Switzerland, Tunisia, Turkey.

**12. Phlebotomus (Larroussius) tobbi Adler & Theodor in Adler, Theodor & Lourie, 1930**

**Selected published records.**Perfil’ev (1966: 296–300); Lewis (1982: 162–163); Artemiev and Neronov (1984: p. 101–102); Wagner (1990: 17); Melaun et al. (2014: 2298).

**Distribution.** Albania, Armenia, Azerbaijan, Bosnia and Herzegovina, Croatia, Cyprus, Georgia, Greece, Iran, Israel, Italy, Jordan, Lebanon, Palestine, Serbia, Sicily, Slovenia, Syria, Turkey.

###### Subgenus Adlerius Nitzulescu, 1931

**13. Phlebotomus (Adlerius) balcanicus Theodor, 1958**

**Selected published records**. Perfil’ev (1966: 316–317, as *P.chinensisbalcanicus* Theodor, 1958); Lewis (1982: 165, as species); Artemiev and Neronov (1984: 114, as species); Wagner (1990, 2013: 17, as species); Melaun et al. (2014: 2298, as species).

**Distribution**. Albania, Azerbaijan, Bosnia and Herzegovina, Bulgaria, Caucasus, Crimea, Croatia, Georgia, Greece, Hungary, Iran (north-western), Macedonia, Romania, Serbia, Turkey, Ukraine.

**14. Phlebotomus (Adlerius) brevis Theodor & Mesghali, 196**4

Syn. *Phlebotomuschinensisismailicus* Perfil’ev, 1966

**Published records.**Perfil’ev (1966: 314, as *P.chinensisismailicus* Perfil’ev, 1966); Lewis (1982: 165); Artemiev and Neronov (1984: 115); Wagner (1990, 2013: 17); Melaun et al. (2014: 2298).

**Comments on distribution.**Artemiev and Neronov (1984): only Iran, southern Caucasus, Turkey. Wagner (1990, 2013) listed records from Moldova. Melaun et al. (2014) listed the species from Azerbaijan, Georgia, Greece and Malta.

**15. Phlebotomus (Adlerius) halepensis Theodor, 1958**

**Selected published records.**Perfil’ev (1966: 305–320, recognized 7 subspecies of *Phlebotomuschinensis* Newstead, 1916: *chinensis, simici* Nitzulescu, 1931, *longiductus* Parrot, 1928, *tauriae* Perfil’ev, 1966, *ismailicus* Perfil’ev, 1966, *balcanicus* and *halepensis*). Lewis (1982: 164–168, recognized five subspecies as valid species and supported synonymies proposed by Artemiev (1978, 1980): *tauriae* = *longiductus*, *ismailicus* = *brevis* Theodor & Mesghali, 1964); Artemiev and Neronov (1984: 107–125, discussed taxonomic problems); Wagner (1990: 18, as species); Melaun et al. (2014: 2298, as species).

**Distribution.** Armenia, Azerbaijan, Georgia, Iran, Israel, Kazakhstan, Kirghistan, Russia, Syria, Tajikistan, Turkey, Turkmenistan, Uzbekistan.

**16. Phlebotomus (Adlerius) simici Nitzulescu, 1931**

**Selected published records.**Perfil’ev (1966: 307, as subspecies *chinensissimici*, see *P.halepensis*); Lewis (1982: 168 as species); Artemiev and Neronov (1984: 123, as species); Wagner (1990: 18, as species).

**Distribution.** Armenia, Azerbaijan, Crete, Georgia, Greece, Jordan, Iran, Israel, Kazakhstan, Palestine, Romania, Russia, Syria, Turkey, former Yugoslavia.

##### *Sergentomyia* França & Parrot, 1920

###### Subgenus Sergentomyia s. str.

**17. Sergentomyia (Sergentomyia) dentata Sinton, 1933**

**Selected published records**. Perfil’ev (1966: 337); Lewis (1967: 25); Wagner (1990: 23).

**Distribution.** Azerbaijan, Greece, Iran, Iraq, Kazakhstan, Pakistan, Turkey, Turkmenistan, Uzbekistan.

###### Subgenus Neophlebotomus França & Parrot, 1920

**18. Sergentomyia (Neophlebotomus) pawlowskyi (Perfiliev, 1933)**

**Selected published records.**Perfil’ev (1966: 361–365, originally described the species as a *Phlebotomus*, Lewis (1967) later included it in Sergentomyia (Rondanomyia) Theodor, 1958; this subgenus was synonymized with *Neophlebotomus* (Lewis 1978: 269)); Wagner (1990: 22).

**Distribution.** Afghanistan, Armenia, Azerbaijan, Georgia, Iran, Iraq, Tajikistan, Turkey, Turkmenistan, Uzbekistan.

###### Subgenus Parrotomyia Theodor, 1958

**19. Sergentomyia (Parrotomyia) palestinensis (Adler & Theodor, 1927)**

**Selected published records.** Adler & Theodor (1927) originally described this species in *Phlebotomus*; Lewis (1967) later included it in the genus *Sergentomyia*, which was recognized as well by Perfil’ev (1966: 352–355); Perfil’ev (1968: 318, 326, in subgenus Parrotomyia); Lewis (1978: 264, in subgenus Parrotomyia); Wagner (1990: 25, subgenus Parrotomyia).

**Distribution.** Afghanistan, Armenia, Azerbaijan, Georgia, Iran, Iraq, Israel, Jordan, Palestine, Pakistan, Saudi Arabia. Oriental Region.

#### Psychodinae

##### Mormiini

###### Mormiina

####### *Yomormia* Ježek, 1984

**20. *Yomormiapetrovi* Ježek, 1985**

**Material examined. Armenia**: Tavush Province, Bldan River, below Jukhtakvank monastery and the mineral water factory/plant, Arm 11, 28.viii.2015, 1 ♂, O Ma H leg. SW, NMPC slide 22537.

**Comments.** This species was known only from the original description from Sandanski, Bulgaria (Ježek 1985). Krek (1999) listed this species as Limomormia (Limomormia) petrovi (Ježek 1985: 122–123). New for Armenia.

##### Paramormiini

###### Paramormiina

####### *Clogmia* Enderlein, 1937

**21. *Clogmiaalbipunctata* (Williston, 1893)**

**Material examined. Armenia**: Yerevan, Tigran Mets Avenue, Arm 1, 30.viii.2015, 1 ♂, O leg., HC, NMPC slide 22575.

**Comments.** This is an expansive, often synanthropic circumtropical and circumsubtropical species (Ježek and Goutner 1995; Werner 1997; Wagner et al. 2002; Boumans 2009; Boumans et al. 2009; Ježek et al. 2012; Oboňa and Ježek 2012; Kvifte et al. 2013; Wagner 2013; Humala and Polevoi 2015; Afzan and Belqat 2016; Bejarano and Estrada 2016; Oboňa et al. 2016a; Afzan and Belqat 2016; Cazorla-Perfetti and Moreno 2017). New for Armenia.

####### *Parajungiella* Vaillant, 1972

**21. *Parajungiellaabchazica* Ježek, 1985**

**Material examined. Armenia**: Tavush Province, Bldan River, above Dilijan City, Arm 10, 28.viii.2015, 1♂, O Ma H leg. SW, NMPC slide 22528; Bldan River, below Jukhtakvank monastery and the mineral water factory/plant, Arm 11, 28.viii.2015, 1 ♂, O Ma H leg. SW, NMPC slide 22538.

**Comments.** This species was known by its original description from Saken-narzan, Abkhazia (Ježek 1985: 52–55). New for Armenia.

**22. *Parajungiellamonikae* (Wagner & Joost, 1986), comb. n.**

**Comments.** This species from Armenia was originally described by Wagner and Joost (1986: 112) as *Jungiellamonikae*. Its generic placement was changed to *Parajungiella* Vaillant, 1972 (: 83, originally as subgenus of *Jungiella* Vaillant, 1972) by Ježek (1984: 166). This arrangement was subsequently followed by Krek (1999: 5), Kvifte et al. (2011), Salmela et al. (2014), and Omelková and Ježek (2017).

####### *Paramormia* Enderlein, 1935

######## Subgenus Duckhousiella Vaillant, 1972

**23. Paramormia (Duckhousiella) ustulata (Walker, 1856)**

**Material examined. Armenia**: Lori Province, tributary of Dzoraget River, above Pushkin village, Arm 27, 3.ix.2015, 1M, O Ma H leg. SW, NMPC slide 22541; Ararat Province, above the confluence of Azat and Gekhard rivers, Arm 17, 31.viii.2015,1 ♂, O Ma H leg. SW, NMPC slide 22582; Lori Province, tributary of Pambak River, at the H24 road switch-backs , Arm 22, 1.ix.2015, 1♀, O Ma H leg. SW, NMPC slide 22589; Kotayk Province, Hrazdan River, below Hrazdan Reservoir, Arm 6, 27.viii.2015, 1 ♂, O Ma H leg. SW, NMPC slide 22597.

**Comments.** This is a widespread species or complex of species occurring in the Holarctic region. Ježek and Yağci (2005) listed occurrences from the following countries: Afghanistan, Algeria, the Azores, Belgium, Bosnia-Herzegovina, Bulgaria, the Canary Islands, China, Corsica, Czech Republic, Denmark, France, Germany, Great Britain, Greece, Hungary, Iran, Ireland, Israel, Italy, Macedonia, Madeira, Mongolia, Morocco, the Netherlands, Poland, Romania, Sardinia, Serbia, Slovakia, Slovenia, Spain, Sweden, Switzerland, Tunisia, Turkey and the USA. It has also been recorded from Finland (Salmela et al. 2014) and Mallorca (Kvifte et al. 2016). New for Armenia.

######## Subgenus Paramormia s. str.

**24. Paramormia (Paramormia) fratercula (Eaton, 1893)**

**Comments.**Eaton (1893: 128) described this species as *Pericomafratercula* from Great Britain. Ježek (1984: 162, 163) considered it to be a Paramormia (Paramormia), within the tribe Paramormiini, and Wagner (1990: 49) placed it in *Parmormiafratercula* in tribe Telmatoscopini.

**Distribution.** Denmark, Germany, Great Britain, Hungary, the Netherlands, Sweden (Wagner 1990, 2013). As first record from Armenia by Wagner and Joost (1986: 111).

**25. Paramormia (Paramormia) polyascoidea (Krek, 1971)**

**Material examined. Armenia**: Shirak Province, tributary of Akhurian River, at Torosgyugh village, Arm 25, 3.ix.2015, 1 ♂, O Ma H leg. SW, NMPC slide 22600.

**Comments.** This is a common European and west Siberian species known from Austria, Bosnia and Herzegovina, Czech Republic, Estonia, Finland, Germany, Poland, Abkhazia, and Russia (Novosibirsk region) (Salmela and Piirainen 2005; Ježek and Omelkova 2012; Salmela et al. 2014). New for Armenia.

####### *Peripsychoda* Enderlein, 1935

**26. *Peripsychodaauriculata* (Haliday in Curtis, 1839)**

**Material examined. Armenia**: Tavush Province, Bldan River, below Jukhtakvank monastery and mineral water factory/plant, Arm 11, 28.viii.2015, 1 ♂, O Ma H leg. SW, NMPC slide 22536; Tavush Province, Bldan River, above Dilijan City, Arm 10, 28.viii.2015, 1 ♂, O Ma H leg. SW, NMPC slide 22527.

**Comments.** This is a large, black, conspicuous European and Transcaucasian species that is very common in lowlands to hilly regions. For detailed distributional data, see Wagner (1990, 2013) and Ježek (2004). New for Armenia.

##### Psychodini

###### *Logima* Eaton, 1904

**27. *Logimaalbipennis* (Zetterstedt, 1850)**

**Material examined. Armenia**: Ararat Province, Gekhard River, at Gerghard monastery (parking place), Arm 14, 30.viii.2015, 1 ♀, O Ma H leg. SW, NMPC slide 22574. **Azerbaijan**: Qabala district, S of Durca, light trap near stream, tributary of Qaraschay River, Aze 4, 30.v.2017, 1 ♀, H leg. LT, NMPC slide 24175.

**Comments.** This is a cosmopolitan species, very common from lowlands to mountains.

**Distribution.** In Europe, it is known from Austria, Azores, Belgium, Bosnia and Herzegovina, Bulgaria, Czech Republic, Denmark, Finland, France, Germany, Great Britain, Greece, Hungary, Ireland, Italy, Luxemburg, Madeira, the Netherlands, Norway, Poland, Portugal, Romania, Russia, Sardinia, Serbia, Slovakia, Slovenia, and Sweden. In Asia from Afghanistan, China, India, Japan, North Korea, Syria and Turkey. In Afrika, from Algeria, the Canary Islands, Gambia, South Africa, Tunisia; also from Australia, New Zealand, South America, USA; Campbell Island, Juan Fernandez Island, Kerguelen Island, Macquarie Island (Ježek and Yağci 2005; Kvifte 2012; Afzan and Belqat 2016). New for Armenia and Azerbaijan.

**28. *Logimasatchelli* (Quate, 1955)**

**Material examined. Azerbaijan**: Qabala district, S of Durca, light trap near stream, tributary of Qaraschay River, Aze 4, 30.v.2017, 1 ♀, H leg. LT, NMPC slide 24174.

**Distribution.** This is a common Holarctic species. In Europe, known from e.g. Austria, Czech Republic, Ireland, Italy, Norway, Russia, Slovakia, Slovenia, Sweden, the Netherlands, Ukraine and the former Yugoslavia; Canada, USA (Ježek and Goutner 1995; Svensson 2009; Kvifte et al. 2011; Ježek et al. 2017). New for Azerbaijan.

###### Psychoda Latreille, 1796

**29. *Psychodauniformata* Haseman, 1907**

**Material examined. Armenia**: Kotayk Province, tributary of Marmarik River, above Meghradzor, behind railway, Arm 7, 27.vii.2015, 1 ♀, O Ma H leg. SW, NMPC slide 22601; Lori Province, tributary of Dzoraget River, above Pushkin village, Arm 27, 3.ix.2015, 1 ♀, O Ma H leg. SW, NMPC slide 22542.

**Comments.** This is a Holarctic species, recorded from Europe (Austria, Czech Republic, Italy, Slovakia, Slovenia, Greece), Turkey, Iran, Israel, Mongolia, Morocco and the USA (Ježek and Omelková 2012; Oboňa and Ježek 2014; Afzan and Belqat 2016). New for Armenia.

###### *Psychodocha* Ježek, 1984

**30. *Psychodochacinerea* (Banks, 1894)**

**Material examined. Armenia**: Ararat Province, Gekhard River, at Gerghard monastery (parking place), Arm 14, 30.viii.2015, 1 ♀, O Ma H leg. SW, NMPC slide 22573; Lori Province, tributary of Dzoraget River, above Pushkin village, Arm 27, 3.ix.2015, 1 ♀, O Ma H leg. SW, NMPC slide 22543.

**Distribution.** This is a very common cosmopolitan species, in Europe, it is known from Austria, Azores, Belgium, Bosnia and Herzegovina, Bulgaria, Canary Islands, Cyprus, Czech Republic, Denmark, Finland, France, Finland, Germany, Great Britain, Greece, Hungary, Ireland, Italy (including Sardinia), Madeira, the Netherlands, Norway, Poland, Romania, Russia, Serbia, Slovakia, Slovenia, Spain, Sweden, Switzerland, Turkey and Ukraine. In Asia, from Abkhazia, Afghanistan, Iran, and Israel. In Africa, from Algeria, Morocco, South Africa, and Tunisia. In the Americas, from Argentina; Brazil, Canada, Chile, Jaun Fernandéz Island, Puerto Rico, USA. Also known from Australia and New Zealand (Krek 1985; Ježek and Goutner 1995; Ježek and Yağci 2005; Kvifte et al. 2011; Kvifte 2012; Wagner 1990, 2013; Salmela et al. 2014; Afzan and Belqat 2016; Ježek et al. 2017). New for Armenia.

###### *Tinearia* Schellenberg, 1803

**31. *Tineariaalternata* (Say, 1824)**

**Material examined. Armenia**: Lori Province, tributary of Dzoraget River, above Pushkin village, Arm 27, 3.ix.2015, 1 ♂, O Ma H leg. SW, NMPC slide 22540; Lori Province, tributary of Aghstev River, above M8 road at Lermontov village, Arm 20, 31.viii.2015,1 ♀, O Ma H leg. SW, NMPC slide 22559; Ararat Province, above the confluence of Azat and Gekhard rivers, Arm 17, 31.viii.2015, 1 ♀, O Ma H leg. SW, NMPC slide 22581; Kotayk Province, Marmarik district, Marmarik near road H28, Arm 3, 26.viii.2015, 1 ♀, O Ma H leg. LT, NMPC slide 22583; Ararat Province, Azat River, at Lanjazat village, Arm 19, 31.viii.2015, 1 ♀, O Ma H leg. SW, NMPC slide 22595; Kotayk Province, Marmarik district, Marmarik Secondary School, Arm 2, 27.viii.2015, 1 ♂, O Ma H leg. BH, NMPC slide 22608. **Azerbaijan**: Khizi district, S of Sitalçay, wetland/pasture near Sumgayit bypass highway, Aze 1, 26.v.2017, 1 ♀, H leg. SW, NMPC slide 24173.

**Distribution.** This is a cosmopolitan species that is generally very common. In Europe, it is known from Austria, Balearic Islands, Belgium, Bulgaria, Crete, Croatia, Cyprus, Czech Republic, Denmark, Finland, France, Germany, Great Britain, Greece, Hungary, Ireland, Italy, Madeira, Norway, Poland, Romania, Sardinia, Slovenia, Spain, Sweden, Switzerland, the Netherlands, and Ukraine. In Asia, from Afghanistan and UAE. In Africa from Algeria, Cape Verde Islands, Canary Islands, D.R. Congo, Egypt, Gambia, Ghana, Morocoo, Nigeria, the Seychelles, Socotra Island, South Africa, Tanzania, and Tunisia. In Asia from Bangladesh, Borneo, Philippines, India, Jamaica, Japan, Malaysia, Mongolia, North Korea, Ryukyu Islands, Taiwan. From North and South America, including Panama, Puerto Rico, and Trinidad. Also from Australia, Hawaii, and from Micronesia, Macquarie Islands, New Zealand, and Samoa. (Ježek 1981; Ježek and van Harten 1996; Kvifte et al. 2011; Kvifte 2012; Ježek and Tkoč 2012; Wagner 2013; Afzan and Belqat 2016; Ježek et al. 2017). New for Armenia and Azerbaijan.

##### Pericomaini

###### *Joostiella* Vaillant, 1983

**32. *Joostiellacaucasica* Vaillant, 1983**

**Comments.** The genus and species, originally described from near the Baksar River, Zlohl in the central Caucasus, were published by Vaillant (1983: 335). Wagner (1986: 87) later recorded this species from Turkey and Armenia, and additional records from Armenia were published by Wagner and Joost (1986: 111).

###### *Pericoma* Walker, 1856

####### Subgenus Pachypericoma Vaillant, 1978

**33. Pericoma (Pachypericoma) blandula Eaton, 1893**

**Material examined. Armenia**: Tavush Province, tributary of Gosh River, spring area at parking place and cafeteria, Arm 28, 4.ix.2015, 1 ♂, O Ma H leg. SW, NMPC slide 22546; Lori Province, tributary of the Pambak River, at the H24 road switch-backs, Arm 22, 1.ix.2015, 1 ♂, O Ma H leg. SW, NMPC slide 22588; Tavush Province, tributary of Aghstev River, above Teghut town, Arm 12, 29.viii.2015, 1 ♂, O Ma H leg. SW, NMPC slide 22592; Lori Province, small brook, in valley at road H23 to Pushkin Pass, Arm 26, 3.ix.2015, 1 ♂, O Ma H leg. SW, NMPC slide 22594; Lori Province, Zamanlu River, a tributary of Pambak River, at Vahagnadzor town, Arm 21, 1.ix.2015, 1 ♂, O Ma H leg. SW, NMPC slide 22607. **Azerbaijan**: Lankaran district, SW of Lankaran, stream with woody vegetation, tributary of Lankaran River, Aze 3, 3.vi.2017, 2 ♂, H leg. SW, NMPC slides 24171 and 24172.

**Distribution.** This species is widespread in Europe, known from Austria, Belgium, Bosnia and Herzegovina, Bulgaria, Croatia, Czech Republic, Denmark, European Turkey, Finland, France, Germany, Great Britain, Greece, Hungary, Ireland, Italy, Macedonia, Montenegro, Norway, Poland, Romania, European Russia, Sardinia, Serbia, Slovakia, Slovenia, Spain, Sweden, Switzerland, the Netherlands and Ukraine. It is also recorded in Transcaucasia, Tunisia, and Morocco (Ježek 2004; Kvifte et al. 2011, 2013; Ježek et al. 2017). New for Armenia and Azerbaijan.

####### Subgenus Pericoma s. str.

**34. *Pericomabosniaca* Krek, 1966**

**Published records.**Krek (1966: 249, 250; 1967a: 256, 257, 258; 1967b: 316; 1970: 98; 1972: 63; 1974: 60; 1979a: 1807, 1809; 1982: 154; 1985: 155, 179; 1999: 378, 379, as Pericoma (Leptopericoma) bosniaca Krek, 1967); Krek et al. (1976: 29); Mučibabič et al. (1984: 64); Vaillant (1978: 211, 219, pl. 66 fig 6, pl. 67 figs 7, 8); Wagner (1990: 38, as *bosnica* Krek, 1967); Wagner and Joost (1988: 30, the same).

**Material examined. Armenia**: Ararat Province, small tributary of Azat River, waterfall at road, Arm 16, 31.viii.2015, 2 ♂, O Ma H leg. SW, NMPC slides 22551 and 22552.

**Distribution.** Bosnia and Herzegovina, Bulgaria, Montenegro, Serbia, Macedonia. New for Armenia.

**35. *Pericomabunae* Krek, 1979**

**Published records.**Krek (1979b: 127, 128, 129); Wagner (1990: 38, journal reference error); Krek (1999: 376, 377, as Pericoma (Leptopericoma) bunae Krek, 1979).

**Material examined. Azerbaijan**: Quba district, Xinaliq village, mountain stream, Aze 2, 27.v.2017, 1 ♂, H leg. SW, NMPC slide 24170.

**Distribution**: This species is known from Bosnia and Herzegovina, as well as Montenegro. New for Azerbaijan.

**36. *Pericomaexquisita* Eaton, 1893**

**Material examined. Armenia**: Shirak Province, tributary of Akhurian River, in valley below road from above Amasia town, Arm 24, 2.ix.2015, 1 ♂, O Ma H leg. SW, NMPC slide 22530; Tavush Province, tributary of Gosh River, spring area at the parking place and cafeteria, Arm 28, 4.ix.2015, 1 ♂, O Ma H leg. SW, NMPC slide 22547; Ararat Province, Gekhard River, below Garni Temple, Arm 15, 31.viii.2015, 1 ♂, O Ma H leg. SW, NMPC slide 22578; Ararat Province, above the confluence of Azat and Gekhard rivers, Arm 17, 31.viii.2015, 1 ♂, O Ma H leg. SW, NMPC slide 22587; Kotayk Province, Hrazdan River, above Solak town, Arm 5, 27.viii.2015, 1 ♂, O Ma H leg. SW, NMPC slide 22599.

**Distribution.** This species is widespread in Europe, North Africa (Algeria, Morocco, and Tunisia), and Transcaucasia (Armenia; Wagner 1981: 56). In Europe, it is known from Albania, Austria, Belgium, Bosnia and Herzegovina, Bulgaria, Czech Republic, Crete, Croatia, France, Germany, Great Britain, Greece, Hungary, Ireland, Italy, Macedonia, Montenegro, Poland, Serbia, Slovakia, Slovenia, Spain and Ukraine (Ježek 2004, 2009; Kvifte et al. 2013; Wagner 2013; Oboňa and Ježek 2014; Afzan and Belqat 2016; Ježek et al. 2017).

**37. *Pericomaplatystyla* Wagner, 1986**

**Material examined. Armenia**: Ararat Province, small tributary of Azat River, waterfall at road, Arm 16, 31.viii.2015, 1 ♂, O Ma H leg. SW, NMPC slide 22553.

**Comments.**Wagner (1986: 84) described this species from Turkey and Greece. New for Armenia.

###### *Pneumia* Enderlein, 1935

**38. *Pneumiacanescens* (Meigen, 1804)**

**Material examined. Armenia**: Shirak Province, tributary of Akhurian River, in valley below road from above Amasia town, Arm 24, 2.ix.2015, 1 ♂, O Ma H leg. SW, NMPC slide 22534; Tavush Province, tributary of Gosh River, spring area at parking place and cafeteria, Arm 28, 4.ix.2015, 1 ♂, O Ma H leg. SW, NMPC slide 22534.

**Comments.** This is a common European and western Siberian species. In Europe it is known Austria, Belgium, Great Britain, Czech Republic, Denmark, European Turkey, France, Germany, Greece, Hungary, Slovakia, Sweden, the Netherlands and the former Yugoslavia. In Asia, it is known from Turkey, Kyrgyzstan, Afghanistan and China (Ježek 2006; Omelková and Ježek 2012; Ježek et al. 2013). New for Armenia.

**39. *Pneumiajoosti* (Wagner, 1981)**

**Material examined. Armenia**: Kotayk Province, tributary of Marmarik River, above recreation centre, Arm 8, 27.viii.2015, 1 ♂, O Ma H leg. SW, NMPC slide 22606; Kotayk Province, Hrazdan River, above Solak town, Arm 5, 27.viii.2015, 1 ♂, O Ma H leg. SW, NMPC slide 22598; Lori Province, small brook, in valley at road H23 to Pushkin Pass, Arm 26, 3.ix.2015, 1 ♂, O Ma H leg. SW, NMPC slide 22593; Lori Province, small steppe brook, tributary of Dzoraget River, Arm 23, 2.ix.2015, 1 ♂, O Ma H leg. SW, NMPC slide 22591; Kotayk Province, Marmarik River, below Hankavan, Arm 4, 26.viii.2015, 1 ♂, O Ma H leg. SW, NMPC slide 22586; Lori Province, tributary of Aghstev River, above M8 road at Lermontov village, Arm 20, 31.viii.2015, 6 ♂, O Ma H leg. SW, NMPC slides 22561–22566; Shirak Province, tributary of Akhurian River, in valley below road from above Amasia town, Arm 24, 2.ix.2015, 3 ♂, O Ma H leg. SW, NMPC slides 22531–22533.

**Comments.**Wagner (1981) described this species from Armenia as *Satchelliellajoosti*.

**Distribution.** This species is known only from Armenia and Transcaucasus (Wagner 1981).

**40. *Pneumiapilularia* (Tonnoir, 1940)**

**Material examined. Armenia**: Kotayk Province, tributary of the Marmarik River, above recreation centre, Arm 8, 27.viii.2015, 3 ♂, O Ma H leg. SW, NMPC slides 22549, 22605 and 22579; Ararat Province, small tributary of Azat River, waterfall at road, Arm 16, 31.viii.2015, 1 ♂, O Ma H leg. SW, NMPC slide 22550; Lori Province, tributary of Aghstev River, above M8 road at Lermontov village, Arm 20, 31.viii.2015, 1 ♂, O Ma H leg. SW, NMPC slide 22560; Ararat Province, Gekhard River, at Gerghard monastery (parking place), Arm 14, 30.viii.2015, 1 ♂, O Ma H leg. SW, NMPC slide 22572; Ararat Province, above the confluence of Azat and Gekhard rivers, Arm 17, 31.viii.2015, 1 ♂, O Ma H leg. SW, NMPC slide 22580; Ararat Province, small tributary of Gekhard River, crossroad at factory, Arm 18, 31.viii.2015, 1 ♂, O Ma H leg. SW, NMPC slide 22603; Gegharkunik Province, Dzknaget River, at Sevan Lake and M14 road, Arm 13, 29.viii.2015, 1 ♂, O Ma H leg. SW, NMPC slide 22604.

**Comments.** This species is distributed throughout almost all of Europe, including Spain, the British Isles and Scandinavia. It is also known from Algeria, Morocco, the Central Caucasus (Terskol, Russia) and Tajikistan, but it is relatively sporadic there (Wagner 1990; Vaillant and Joost 1983; Ježek 2004; Ježek et al. 2014). New for Armenia.

**41. *Pneumianubila* (Meigen, 1818)**

**Material examined. Armenia**: Tavush Province, Bldan River, above Dilijan City, Arm 10, 28.viii.2015, 1 ♂, O Ma H leg. SW, NMPC slide 22526; Shirak Province, tributary of Akhurian River, in valley below road from above Amasia town, Arm 24, 2.ix.2015, 1 ♂, O Ma H leg. SW, NMPC slide 22529; Tavush Province, Bldan River, below Jukhtakvank monastery and mineral water factory/plant, Arm 11, 28.viii.2005, 1 ♂, O Ma H leg. SW, NMPC slide 22535; Lori Province, tributary of Dzoraget River, above Pushkin village, Arm 27, 3.ix.2015, 1 ♂, O Ma H leg. SW, NMPC slide 22539; Tavush Province, tributary of Gosh River, spring area at parking place and cafeteria, Arm 28, 4.ix.2015, 1 ♂, O Ma H leg. SW, NMPC slide 22544; Kotayk Province, tributary of Marmarik River, above the recreation centre, Arm 8, 27.viii.2015, 1 ♂, O Ma H leg. SW, NMPC slide 22548; Lori Province, tributary of Aghstev River, above M8 road at Lermontov village, Arm 8, 31.viii.2015, 1 ♂, O Ma H leg. SW, NMPC slide 22567; Ararat Province, Gekhard River, at Gerghard monastery (parking place), Arm 14, 30.viii.2015, 1 ♂, O Ma H leg. SW, NMPC slide 22571; Lori Province, small steppe brook, tributary of Dzoraget River, Arm 23, 2.ix.2015, 1 ♂, O Ma H leg. SW, NMPC slide 22590; Kotayk Province, Hrazdan River, below Hrazdan Reservoir, Arm 6, 27.viii.2015, 1 ♂, O Ma H leg. SW, NMPC slide 22596.

**Distribution.** This is a very common species, which is recorded from throughout Europe and the Canary Islands. In Europe, it is known from Austria, Belgium, Bosnia and Herzegovina, Bulgaria, Croatia, Czech Republic, Denmark, Finland, France, Georgia, Germany, Great Britain, Greece, Hungary, Ireland, Italy, Luxembourg, Macedonia, Montenegro, Poland, Romania, Sardinia, Serbia, Slovakia, Slovenia, Spain, Switzerland, the Netherlands and Ukraine (Wagner 1981; Ježek and Goutner 1995; Krek 1999; Ježek 2002; Kvifte et al. 2013; Ježek et al. 2016). New for Armenia.

###### *Saraiella* Vaillant, 1973

**42. *Saraiellaressliressli* Wagner, 1981**

**Material examined. Armenia**: Ararat Province, small tributary of Azat River, waterfall at road, Arm 16, 31.viii.2015, 5 ♂, O Ma H leg. SW, NMPC slides 22554–22558; Lori Province, tributary of Aghstev River, above M8 road at Lermontov village, Arm 20, 31.viii.2015, 2 ♂, O Ma H leg. SW, NMPC slides 22569 and 22570; Tavush Province, Lake Parz Lich and its tributary, lake Parz Lich, Arm 9, 28.8.2015, 1 ♂, O Ma H leg. SW, NMPC slides 22576 and 22577; Ararat Province, confluence of Azat and Gekhard rivers, Arm 17, 31.viii.2015, 2 ♂, O Ma H leg. SW, NMPC slides 22584 and 22585; Ararat Province, small tributary of Gekhard River, crossroad at factory, Arm 18, 31.viii.2015, 1 ♂, O Ma H leg. SW, NMPC slide 22602. **Azerbaijan**: Quba district, Xinaliq village, mountain stream, Aze 2, 27.v.2017: 1 ♂, H leg. SW, NMPC slide 24169.

**Comments.** This species was described by Wagner (1981: 48) from northern Iran, from material collected at Veyshahr near Nowshahr on the Caspian Sea. Wagner and Joost (1983: 100) published central Caucasus record from Terskol, Russia. Ježek (1990: 27–30) published a record of a subspecies, *Saraiellaresslimontana*, from Kerman province in south-eastern Iran. New for Armenia and Azerbaijan.

###### *Thornburghiella* Vaillant, 1973

**43. *Thornburghiellaveve* Oboňa & Ježek, 2017**

**Published record. Armenia**: Oboňa et al. 2017a (Lori Province near Dzoraget village, tributary of Pambak River), 3 ♂, NMPC type material catalogue no. 34708-34710, inventory no. 22620-22622.

###### *Ulomyia* Walker, 1856

**44. *Ulomyiacognata* (Eaton, 1893)**

**Material examined. Armenia**: Lori Province, tributary of Aghstev River, above M8 road at Lermontov village, Arm 20, 31.viii.2015, 1 ♂, O Ma H leg. SW, NMPC slide 22568.

**Distribution.** This is a common European species known from Austria, Czech Republic, Finland, France, Germany, Great Britain, Italy, Lithuania, Poland, Slovakia, Slovenia and Ukraine (Ježek and Omelková 2012; Salmela et al. 2014; Ježek et al. 2017). It is known from the central Caucasus at Terskol, Russia (Vaillant and Joost 1983: 100). New for Armenia.

## Discussion

According to Oboňa et al. (2016b, 2017b) and Hrivniak et al. (2018), as compared to many European countries, Transcaucasia (Georgia, Armenia, Azerbaijan) is insufficiently investigated for flies of the families Anisopodidae, Bibionidae, Dixidae, Drosophilidae, Limoniidae, Pediciidae, Ptychopteridae, and Scatopsidae. Only 17 species of Psychodidae are known for Armenia and 18 species are known from Azerbaijan (Perfil’ev 1966; Lewis 1982; Artemiev and Neronov 1984; Vaillant 1972; Wagner 1981, 1990, 2013; Wagner and Joost 1986; Wagner et al. 2002; Melaun et al. 2014; Oboňa et al. 2017a). In the present paper, we include 35 recent species (18 of them newly recorded) from Armenia. Included are 11 species of Phlebotominae and 24 species of Psychodinae. Similarly, our checklist of moth flies from Azerbaijan includes 24 species, of which 6 are newly recorded. The species recorded include 18 species of Phlebotominae and 6 of Psychodinae. Our results certainly represent only a small part of the Armenian and Azerbaijan psychodid fauna. Finding common and widespread species such as Paramormia (Duckhousiella) ustulata, *Psychodochacinerea*, *Tineariaalternata*, Pericoma (Pachypericoma) blandula, *Pneumiacanescens*, *Pneumianubila*, or invasive species such as *Clogmiaalbipunctata*, for the first time from these countries shows that the psychodid fauna is still poorly known.

Knowledge of species distribution is important for studying biogeography and making effective conservation actions. This checklist will provide a baseline for further studies and for initiation of concerted conservation actions in Armenia and Azerbaijan. No doubt that future collecting in Georgia and Azerbaijan, done with the support of the International Visegrad Fund (project No. 21810533), will yield additional faunistic novelties of interest, as shown by similar studies in this region of other dipteran families (e.g. Oboňa et al. 2016b, 2017b; Negrobov et al. 2017; Starý et al. 2017; Hrivniak et al. 2018).
